# Association between fluid infusions and the recovery from acute kidney injury in patients administered liposomal amphotericin B: a nationwide observational study

**DOI:** 10.1080/0886022X.2022.2036618

**Published:** 2022-02-16

**Authors:** Masato Tashiro, Yoko Obata, Takahiro Takazono, Yuki Ota, Tomotaro Wakamura, Yui Shiozawa, Ai Tsuyuki, Taiga Miyazaki, Tomoya Nishino, Koichi Izumikawa

**Affiliations:** a Department of Infectious Diseases, Nagasaki University Graduate School of Biomedical Sciences, Nagasaki, Japan; b Nagasaki University Infection Control and Education Center, Nagasaki University Hospital, Nagasaki, Japan; c Department of Nephrology, Nagasaki University Hospital, Nagasaki, Japan; d Department of Respiratory Medicine, Nagasaki University Hospital, Nagasaki, Japan; e Department of Nephrology, Sasebo City General Hospital, Nagasaki, Japan; f Medical Affairs Division, Sumitomo Dainippon Pharma Co., Ltd., Tokyo, Japan; g Deloitte Tohmatsu Consulting LLC, Tokyo, Japan

**Keywords:** Liposomal amphotericin B, acute kidney injury, AKI recovery, fluid infusion, observational study

## Abstract

Acute kidney injury (AKI) often develops during the administration of liposomal amphotericin B (L-AMB), a broad-spectrum antifungal drug. However, clinical recovery approaches for AKI patients administered L-AMB are not well established. This retrospective analysis used the data obtained from hospitals throughout Japan. AKI was defined as *a* ≥ 1.5-fold increase within 7 days or ≥0.3 mg/dL increase within 2 days in serum creatinine. AKI recovery was defined as a return to creatinine levels below or equal to those recorded before AKI onset. Ninety patients were assessed for recovery from AKI as per the three stages. The incidence of recovery from AKI regardless of its stage was higher, though not significant, in patients administered ≥10 mL/kg/day fluid for 7 consecutive days from AKI onset (63%) than in those who did not (35%, *p* = 0.053). However, if limited to AKI stage 1 patients, the former group had a significantly higher incidence of recovery (91%) than the latter group (50%, *p* = 0.017), even after adjusting for confounding factors (odds ratio: 10.135, 95% confidence interval: 1.148–89.513, *p* = 0.037). The daily fluid volume administered during the 7 consecutive days from AKI onset positively correlated with the recovery from AKI of all stages (*p* = 0.043). Daily consecutive fluid infusion from AKI onset may be associated with recovery from stage 1 AKI in patients administered L-AMB, with daily fluid volume positively correlating with the incidence of AKI recovery.

## Introduction

Invasive fungal infections frequently occur in immunocompromised and critically ill patients and are associated with high rates of morbidity and mortality [1–5]. Amphotericin B is a broad-spectrum antifungal drug that covers clinically relevant yeasts and molds that cause mycoses, such as aspergillosis, candidiasis, cryptococcosis, and mucormycosis [6]. However, the use of amphotericin B has been limited because of its high incidence of toxicities, including nephrotoxicity, liver disorders, and hypokalemia [6]. Liposomal amphotericin B (L-AMB), which encapsulates amphotericin B in a liposomal membrane, was developed to reduce the toxicity of amphotericin B while maintaining its antifungal activity [6]. However, this specific liposomal formulation causes reduced tissue distribution in the kidneys and drug-associated nephrotoxicity, including excessive renal vasoconstriction and renal tubular damage. [7–9]. Despite the reduced nephrotoxicity, physicians are reluctant to prescribe L-AMB, especially to patients with renal failure, because of the risk of acute kidney injury (AKI) [10,11].

Several studies have reported that preexisting renal function, pretreatment and concomitant use of nephrotoxic or antifungal drugs, and L-AMB dosing are associated with AKI in patients administered L-AMB [10–12]. Based on these findings, if these factors are carefully considered, the occurrence of renal dysfunction could be prevented in patients administered L-AMB. However, a study conducted in a single facility revealed that a high volume of fluid infusions one week before and one week after the initiation of L-AMB treatment was not associated with AKI recovery [10]. Although this study suggests that fluid infusion does not contribute to AKI recovery, it appears that the timing, duration, and volume of fluid infusion must still be further scrutinized in a nationwide study. On the basis of claims and laboratory data obtained from hospitals throughout Japan, we identified patients who developed AKI following L-AMB treatment. Thereafter, we investigated the association of fluid infusion before and from the onset of AKI with AKI recovery outcomes and changes in creatinine levels after AKI onset.

## Materials and methods

### Ethics

This study was conducted in accordance with the principles of the Declaration of Helsinki. The data herein were anonymously processed by the database provider (Medical Data Vision Co., Ltd.) in accordance with the Act on the Protection of Personal Information of Japan and other related regulations. The study was approved by the Nagasaki University School of Medicine Research Ethics Committee (approval number 18033038-5). For the usage of un-linkable de-identified data, informed consent was waived by Nagasaki University School of Medicine Research Ethics Committee according to the Japanese Ethical Guidelines for Medical and Health Research Involving Human Subjects by the Ministry of Education, Culture, Sports, Science, and Technology and the Ministry of Health, Labor, and Welfare of Japan.

### Data source

This was a retrospective, multicenter, observational study based on electronic medical information data obtained between April 2008 and January 2018. This database, provided by Medical Data Vision Co., Ltd. (MDV), consisted of medical fee reimbursement claims and clinical laboratory results from 345 Japanese hospitals operated on the Diagnosis Procedure Combination (DPC) system. The database included the following baseline patient information: age, sex, diagnosis, and comorbidities at admission, coded using the International Classification of Diseases, 10th Revision (ICD-10). In addition, the dates and dosages of all drugs administered to each patient during hospitalization were recorded. All interventional procedures were decoded using standardized Japanese reimbursement codes. As DPC is an administrative database for inpatients, patient follow-up began on admission day and ended on discharge date, transferred to other hospitals, or death.

### Study design

In line with the selection criteria described by Takazono et al. [12], we identified patients who developed AKI after receiving the first administration of L-AMB. Thereafter, patients who met the following exclusion criteria were removed: (1) age <18 years; (2) zero and/or single serum creatinine record available during the specified periods to determine the development of AKI and baseline creatinine levels; (3) nonzero weight and/or height record unavailable; (4) a mean daily dose of L-AMB of >6 mg/kg/day per body weight; (5) renal replacement therapy performed on the day of or prior to L-AMB treatment initiation; and (6) AKI developed prior to L-AMB treatment initiation. Furthermore, to determine the changes in serum creatinine levels after AKI onset, patients with no recordings of serum creatinine and/or renal replacement therapy during the evaluation period were excluded. For each patient, the evaluation period started on the day after AKI and ended 30 days after AKI, the day of discharge, death, or the day before L-AMB re-administration, whichever came first. Finally, to determine the incidence of 30-day AKI recovery, we excluded patients with no serum creatinine record between 7 days after AKI and the end of the evaluation period, as well as unrecovered patients, if they were discharged within 29 days or re-administered L-AMB within 30 days of AKI onset.

## Definitions

The duration of L-AMB therapy was defined as the time from treatment initiation to discontinuation, with an administration interval of ≥8 days. AKI was defined as a ≥1.5-fold increase within 7 days or ≥0.3 mg/dL increase within 2 days in serum creatinine level [13]. AKI was assigned to one of three stages: stage 1, ≥1.5- to <2-fold increase or ≥0.3 mg/dL increase in Cr; stage 2, ≥2- to <3-fold increase in Cr; stage 3, ≥3-fold increase in Cr, ≥4.0 mg/dL of Cr [13]. AKI recovery was defined as a return to creatinine levels below or equal to those recorded before the onset of AKI, the minimum creatinine level within 7 days or 2 days before the onset of AKI, with ≥1.5-fold increase or ≥0.3 mg/dL increase, respectively. For the interventions investigated, the daily fluid infusion was determined by daily volume of extracellular replacement fluid per weight, such as saline and balanced crystalloid solutions (the equilibrium buffer solutions contained sodium lactate, sodium acetate, or sodium hydrogen carbonate), excluding parenteral nutrition with chloride. We defined liberal fluid management group as patients infused with daily fluid volume per weight being more than or equal to a specified threshold for a specified period. Patients infused less fluid than the specified threshold on at least one day during the corresponding period were included in the conservative fluid management group. We defined the baseline threshold for daily fluid volume per weight as ≥10 mL/kg/day and fluid administration period as 7 days from the onset of AKI unless otherwise specified. The last nonzero weight recorded on the day of or within 1 year prior to L-AMB treatment initiation was used to determine fluid volume per weight. Discontinuation of L-AMB treatment was defined as the termination of L-AMB administration until or within 3 days after the onset of AKI. Baseline creatinine was defined as the minimum serum creatinine level recorded between 180 days and 7 days before the initiation of L-AMB administration.

With the last nonzero weight and height recorded on the day of or within 1 year prior to L-AMB treatment initiation, the conversion to corresponding estimated glomerular filtration rate (eGFR) was derived using a Japanese-specific formula [14]:

$$\mathrm{eGFR}\;(\mathrm{mL}/\min)=194\;\cdot {\mathrm{creatinine}\;(\mathrm{mg}/\mathrm{dL})}^{-1.094}\cdot {\mathrm{age}\;(\mathrm{years})}^{-0.287}\cdot {1.73(m^{2})}^{-1}\cdot \text{body surface area }(m^{2})\cdot 0.739\;(\text{if feamle})$$

where body surface area (m^2^) was derived as $0.007184\cdot {\text{ weight }(\mathrm{kg})}^{0.425}\;\cdot \;{\mathrm{height}\;(\mathrm{cm})}^{0.725}.$　In addition, the change in minimum creatinine level in a given period after AKI onset was defined as the difference between the creatinine level on the day of AKI onset and the minimum recorded creatinine level in the period, including the day of AKI onset.

Comorbidities and fungal infections were identified using the corresponding ICD-10 codes that were registered in the month of L-AMB treatment initiation. Diabetes mellitus was defined using the ICD10 classification code E10-E14, and chronic kidney disease was defined using the ICD10 code N18. Congestive heart failure was defined using the ICD10 codes described by Quan et al. [15]. Treatment with catecholamine, nephrotoxic drugs, and hypokalemia (<3.5 mEq/L serum potassium) were identified between 7 days before the day of L-AMB treatment initiation and the day before AKI recovery or the end day of AKI recovery evaluation period (30 days after AKI onset, the day of discharge, death, or the day before L-AMB re-administration, whichever came first).

## Statistical analysis

Fisher’s exact test was used to compare the incidence of AKI recovery between the liberal and the conservative fluid management groups. This test was also used to compare the incidence of AKI recovery between patients administered L-AMB who recovered from AKI and patients administered L-AMB who did not recover from AKI. To exclude the effect of confounding factors on AKI recovery, logistic regression analysis was conducted using the occurrence of AKI recovery as the dependent variable. We selected 15 independent variables from the patient characteristics that were considered to be associated with AKI recovery in patients administered L-AMB or that were identified as the factors related to AKI (any stage or stage 2 or 3) in patients administered L-AMB (i.e., treatment with angiotensin-converting enzyme inhibitor/angiotensin receptor blocker [ACE inhibitors/ARB], carbapenem, catecholamine, or immunosuppressants, hypokalemia, and L-AMB average daily dose) [12]. These variables included liberal fluid management, age, sex, comorbidities (diabetes mellitus, congestive heart failure), catecholamine treatment, hypokalemia (<3.5 mEq/L serum potassium), baseline eGFR, L-AMB average daily dose (continuous variable), and treatment with nephrotoxic drugs (vancomycin, aminoglycoside, carbapenem, immunosuppressants, diuretics, or ACE inhibitors/ARB). Variables were subjected to a univariate binomial logistic regression analysis. Variables with a p-value of <0.1 in univariate logistic regression analysis and liberal fluid management were subjected to multivariate logistic regression analysis. Odds ratio (OR), 95% confidence interval (CI), and variance inflation factor (VIF) were calculated. The correlation between the incidence of AKI recovery and fluid infusion volume was determined using the Cochran–Armitage trend test. Finally, Welch’s *t*-test was used to compare the decline in creatinine levels after AKI onset between the liberal fluid management group (from 1 day to 6 days after AKI onset) and the conservative fluid management group. Statistical significance was set at *p*-value of <0.05.

## Results

### Study population and patient characteristics

**Figure 1.**
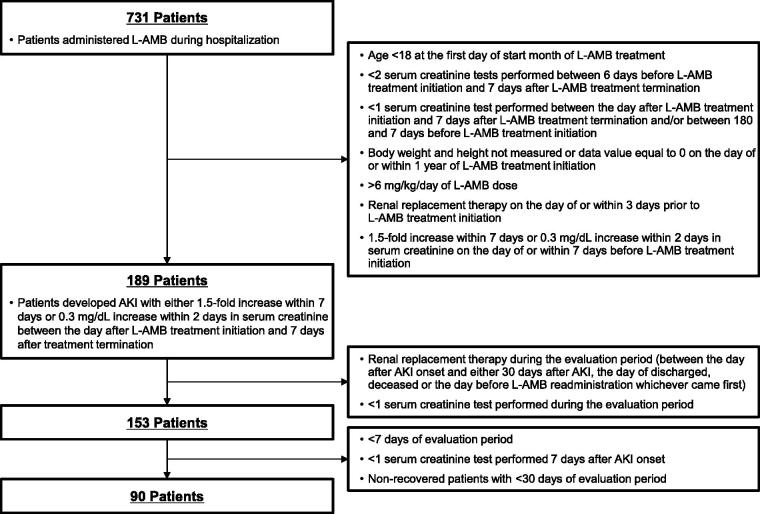
Flow chart for patient selection. AKI: acute kidney injury; L-AMB: liposomal amphotericin B.

By applying the criteria and definitions described in the Materials and methods, 189 patients were identified as having developed AKI following L-AMB administration. Of these patients, we evaluated 90 who were administered L-AMB and developed AKI to assess AKI recovery (Figure 1). As shown in Table 1, 62% of the patients were male and 38% were female. The mean age was approximately 65 years, and the mean body mass index (BMI) was 22.0 ± 4.1 kg/m^2^. The prevalence of comorbidities was as follows: diabetes mellitus, 37%; chronic kidney disease, 8%; and congestive heart failure, 27%. Twenty percent of the patients received treatment with catecholamine, while 80% of the patients suffered from hypokalemia. The mean baseline eGFR was 101.8 ± 42.8 mL/min. More than half of the patients received treatment with several nephrotoxic drugs, such as vancomycin (52%), carbapenem (70%), and diuretics (69%). More than half of the patients were administered L-AMB in the hematology department (56%). Of the fungal infections identified, aspergillosis was the most commonly recognized (29%) among the patients. The mean daily dose was 2.7 ± 0.9 mg/kg/day and the duration of L-AMB administration was approximately 20 days.

**Table 1.** Patient characteristics of liberal and conservative fluid management groups.

Patient characteristics	Overall (*n* = 90)	Liberal fluid management^a^ (*n* = 16, 18%)	Conservative fluid management^b^ (*n* = 74, 82%)	*p*-Value
Sex (%)				
Male	56 (62%)	10 (63%)	46 (62%)	1.000
Female	34 (38%)	6 (38%)	28 (38%)	
Age (years)	64.7 ± 16.2	68.1 ± 14.8	63.9 ± 16.4	0.342
BMI (kg/m^2^)	22.0 ± 4.1	20.3 ± 2.6	22.3 ± 4.3	0.022
Comorbidities, with (vs. without)				
Diabetes mellitus	33 (37%)	5 (31%)	28 (38%)	0.777
Chronic kidney diseases	7 (8%)	1 (6%)	6 (8%)	1.000
Congestive heart failure	24 (27%)	4 (25%)	20 (27%)	1.000
Catecholamine treatment	18 (20%)	3 (19%)	15 (20%)	1.000
Hypokalemia (<3.5 mEq/L serum potassium)	72 (80%)	10 (63%)	62 (84%)	0.081
eGFR at baseline (mL/min)	101.8 ± 42.8	93.4 ± 41.2	103.6 ± 42.9	0.395
Duration between the onset of renal dysfunction and renal recovery (days)	NA	4.4 ± 5.4	4.7 ± 7.9	0.856
L-AMB				
Mean daily dose (mg/day/kg)	2.7 ± 0.9	2.9 ± 1.0	2.6 ± 0.8	0.357
Duration (days)	19.6 ± 19.2	20.6 ± 13.3	19.3 ± 20.3	0.760
Drug treatment, with (vs. without)				
Vancomycin	47 (52%)	7 (44%)	40 (54%)	0.583
Aminoglycoside	16 (18%)	2 (13%)	14 (19%)	0.727
Carbapenem	63 (70%)	11 (69%)	52 (70%)	1.000
Immunosuppressants	13 (14%)	4 (25%)	9 (12%)	0.236
Diuretics	62 (69%)	12 (75%)	50 (68%)	0.767
ACE inhibitors/ARB	17 (19%)	0 (0%)	17 (23%)	0.035
Treatment department (%)				
Hematology	50 (56%)	12 (75%)	38 (51%)	0.102
The internal medicine department, except for hematology	34 (38%)	4 (25%)	30 (41%)	0.394
The surgery department	6 (7%)	0 (0%)	6 (8%)	0.586
Diagnosis (%)				
Fungal infection				
Aspergillosis	26 (29%)	5 (31%)	21 (28%)	0.771
Candidiasis	12 (13%)	1 (6%)	11 (15%)	0.685
Cryptococcosis	6 (7%)	1 (6%)	5 (7%)	1.000
Zygomycosis	1 (1%)	0 (0%)	1 (1%)	1.000
Aspergillosis, Candidiasis	0 (0%)	0 (0%)	0 (0%)	1.000
Aspergillosis, Cryptococcosis	1 (1%)	0 (0%)	1 (1%)	1.000
Aspergillosis, Candidiasis, Cryptococcosis	1 (1%)	0 (0%)	1 (1%)	1.000
Others^c^	21 (23%)	5 (31%)	16 (22%)	0.515
Unknown	18 (20%)	3 (19%)	15 (20%)	1.000
Neutropenia	4 (4%)	1 (6%)	3 (4%)	0.550

ACE inhibitors/ARB: angiotensin-converting enzyme inhibitor/angiotensin receptor blocker; AKI: acute kidney injury; BMI: body mass index; eGFR: estimated glomerular filtration rate; L-AMB: liposomal amphotericin B.

Categorical variables are expressed as frequencies and proportions (%), while continuous variables are expressed as mean ± standard deviation. Welch’s *t*-test was employed to determine the *p*-values for continuous variables, while Fisher’s exact test was applied for categorical variables. Comorbidities and fungal infections were identified using the corresponding ICD-10 codes that were registered in the month of L-AMB treatment initiation. Catecholamine and nephrotoxic drug treatment and hypokalemia were identified between 7 days before the day of L-AMB treatment initiation and the day before AKI recovery or the end day of the AKI recovery evaluation period (30 days after AKI, the day of discharge, death, or the day before L-AMB re-administration, whichever came first).

^a^Daily fluid ≥10 mL/kg infused consecutively for 7 days from AKI onset.

^b^Daily fluid <10 mL/kg infused for at least one day during 7 days from AKI onset.

^c^Others represent unclassified or unspecified mycosis.

Of the 90 patients, 16 (18%) were categorized as a liberal fluid management group. BMI was lower in the liberal fluid management group (20.3 ± 2.6 kg/m^2^) than the conservative fluid management group (22.3 ± 4.3 kg/m^2^, *p* = 0.022). The proportion of patients treated with ACE inhibitors/ARB was lower in the liberal fluid management group (0%) than in the conservative fluid management group (23%, *p* = 0.035). No significant differences were found between the two groups according to sex, age, comorbidities, catecholamine treatment, hypokalemia, eGFR at baseline, average daily dose and duration of L-AMB treatment, treatment department, and diagnosis.

### Association between fluid infusions and recovery from AKI in patients administered L-AMB by AKI stage

As shown in Table 2, in patients with all stages of AKI, the liberal fluid management group had a higher, though not significant, incidence of renal recovery (63%) than in the conservative fluid management group (35%, *p* = 0.053). For patients with AKI stage 1, a significantly higher proportion of patients recovered from AKI in the liberal management group (91%) than in the conservative management group (50%, *p* = 0.017). In patient with AKI stage 2 or 3, the liberal fluid management group did not have a higher incidence of AKI recovery compared to the conservative fluid management group. To exclude the effects of confounding factors on AKI recovery, we conducted logistic regression analysis for patients with all stages of AKI and for patients with AKI stage 1 only. After adjusting for confounding factors, liberal fluid management was not associated with recovery from AKI of all stages (OR: 2.381, 95% CI: 0.701-8.083, *p* = 1.037) (Supplementary Table 1). For AKI stage 1 patients, univariate logistic regression analysis revealed that liberal fluid management and no treatment with vancomycin were associated with AKI recovery (*p* < 0.1) (Table 3). Using these variables in multivariate regression analysis, we found that liberal fluid management was identified as a factor associated with recovery from AKI stage 1 (OR: 10.135, 95% CI: 1.148–89.513, *p* = 0.037) (Table 3). As for other interventions, we examined L-AMB discontinuation until or within 3 days after the onset of AKI; however, no significant difference in the incidence of AKI recovery was observed (19/49, 39%, discontinued; 17/41, 41%, continued, *p* = 0.832).

**Table 2.** Incidence of AKI recovery in patient administered fluid infusion by AKI stage.

	AKI recovery (%)		
AKI stage	Liberal fluid management^a^	Conservative fluid management^b^	*p*-Value
All stages (*N* = 90)	10/16 (63%)	26/74 (35%)	0.053
Stage 1 (*N* = 51)	10/11 (91%)	20/40 (50%)	0.017
Stage 2 (*N* = 24)	0/3 (0%)	5/21 (24%)	1.000
Stage 3 (*N* = 15)	0/2 (0%)	1/13 (8%)	1.000

AKI: acute kidney injury; L-AMB: liposomal amphotericin B.

^a^Daily fluid ≥10 mL/kg infused consecutively for 7 days from AKI onset.

^b^Daily fluid <10 mL/kg infused for at least one day during 7 days from AKI onset.

Fisher’s exact test was performed to determine the *p*-value. The denominator denotes the number of participants.

**Table 3.** Logistic regression analysis of the factors associated with AKI recovery from stage 1 AKI in the liberal and the conservative fluid management groups.

	Univariate regression		Multivariate regression		
Variables	OR (95% CI)	*p*-Value	OR (95% CI)	*p-*Value	VIF
Liberal fluid management^a^ (vs. conservative fluid management^b^)	10.000 (1.169–85.560)	0.036	10.135 (1.148–89.513)	0.037	1.004
Age, ≥65 years (vs. <65 years)	1.231 (0.385–3.937)	0.726			
Sex, male (vs. female)	2.500 (0.770–8.121)	0.127			
Comorbidities, with (vs. without)					
Diabetes mellitus	1.083 (0.345–3.402)	0.891			
Congestive heart failure	1.164 (0.320–4.226)	0.818			
Catecholamine treatment, with (vs without)	1.462 (0.242–8.820)	0.679			
Hypokalemia (<3.5 mEq/L, with [vs without])	0.471 (0.125–1.775)	0.266			
Baseline eGFR, ≥60 mL/min (vs. <60 mL/min)	0.250 (0.027–2.316)	0.222			
L-AMB average daily dose (mg/day/kg, continuous value)	1.284 (0.546–3.021)	0.567			
Drug treatment, with (vs. without)					
Vancomycin	0.375 (0.119–1.185)	0.095	0.369 (0.108–1.262)	0.112	1.004
Aminoglycoside	0.492 (0.115–2.109)	0.340			
Carbapenem	1.167 (0.352–3.862)	0.801			
Immunosuppressants	0.654 (0.144–2.974)	0.582			
Diuretics	1.295 (0.415–4.048)	0.656			
ACE inhibitors/ARB	0.654 (0.144–2.974)	0.582			

ACE inhibitors/ARB: angiotensin-converting enzyme inhibitor/angiotensin receptor blocker; AKI: acute kidney injury; CI: confidence interval; eGFR: estimated glomerular filtration rate; L-AMB: liposomal amphotericin B; OR: odds ratio; VIF: variance inflation factor.

Logistic regression analysis was conducted on AKI stage 1 patients using AKI recovery as the dependent variable. Fifteen independent variables associated with AKI or AKI recovery were subjected to univariate binomial logistic regression analysis. Variables with a *p*-value of <0.1 in univariate logistic regression analysis and liberal fluid management were subjected to multivariate logistic regression analysis. Comorbidities and fungal infections were identified using the corresponding ICD-10 codes that were registered in the month of L-AMB treatment initiation. Catecholamine and nephrotoxic drug treatment and hypokalemia were identified between 7 days before the day of L-AMB treatment initiation and the day before AKI recovery or the end day of the AKI recovery evaluation period (30 days after AKI, the day of discharge, death, or the day before L-AMB re-administration, whichever came first). The OR, 95% CI, and VIF were calculated.

^a^Daily fluid ≥10 mL/kg infused consecutively for 7 days from AKI onset.

^b^Daily fluid <10 mL/kg infused for at least one day for 7 days from AKI onset.

### Association between daily consecutive fluid infusion timing in relation to the onset of AKI and AKI recovery in patients administered L-AMB

We hereafter assessed the relation between fluid management variations and AKI recovery with patients regardless of their AKI stages due to the limited number of subjects. To examine the association between fluid infusion period or timing and AKI recovery, we examined the incidence of AKI recovery in patients who received fluid infusion 2 days or 7 days before or from AKI onset. As shown in Table 4, the incidence of AKI recovery was similar between the liberal fluid management group (7 days before AKI onset) (33%) and the conservative fluid management group (36%). However, liberal fluid management groups (2 days before/from AKI onset) had a slightly higher, though not significant, incidence of AKI recovery (before AKI onset: 50%; from AKI onset: 50%) than respective conservative fluid management groups (before AKI onset: 35%, *p* = 0.247; from AKI onset: 36%, *p* = 0.242) (Table 4).

**Table 4.** Incidence of AKI recovery in patients administered fluid infusion before and after AKI onset.

	AKI recovery (%)			
Fluid infusion period	Liberal fluid management^a^	Conservative fluid management^b^	OR (95% CI)	*p*-Value
For 7 consecutive days before AKI onset (*N* = 84)	3/9 (33%)	27/75 (36%)	0.890 (0.133–4.579)	1.000
For 2 consecutive days before AKI onset (*N* = 90)	14/28 (50%)	22/62 (35%)	1.806 (0.665–4.945)	0.247
For 2 consecutive days from AKI onset (*N* = 90)	13/26 (50%)	23/64 (36%)	1.771 (0.637–4.955)	0.242
For 7 consecutive days from AKI onset (*N* = 90)^c^	10/16 (63%)	26/74 (35%)	3.036 (0.883–11.399)	0.053

AKI: acute kidney injury; CI: confidence interval; OR: odds ratio.

Fisher’s exact test was performed to determine the OR, 95% CI, and *p*-value. The denominator denotes the number of participants.

^a^Daily fluid ≥10 mL/kg infused consecutively for a given period.

^b^Daily fluid <10 mL/kg infused for at least one day during a given period.

^c^Relisted from Table 2.

### Association between the daily volume of fluid infusion and AKI recovery in patients administered L-AMB

We sought to determine the association between the volume of fluid infusions and the incidence of AKI recovery in patients administered L-AMB. As shown in Table 5, we evaluated the incidence of AKI recovery in patients administered daily fluid infusions between ≥5 mL/kg and ≥30 mL/kg consecutively for 2 days or 7 days from AKI onset. In conservative fluid management groups (2 or 7 days from AKI onset), the incidence of AKI recovery remained relatively constant at approximately 39% for all daily fluid volume thresholds. However, for the liberal fluid management group (2 days from AKI onset), a daily volume of ≥25 mL/kg resulted in the highest incidence of AKI recovery (57%), and there was no significant difference between the incidence of AKI recovery and fluid volume increase. For the liberal fluid management group (7 days from AKI onset), higher incidences of AKI recovery were observed when higher daily fluid volumes were administered. Although the number of subjects in the higher fluid volume groups was extremely limited, daily fluid volume was found to be positively correlated with the incidence of AKI recovery (*p* = 0.043, Cochran–Armitage trend test; <5 mL/kg, 17/49, 35%; ≥5 mL/kg and <10 mL/kg, 9/25, 36%; ≥10 mL/kg and <15 mL/kg, 5/8, 63%; ≥15 mL/kg and <20 mL/kg, 1/2, 50%; ≥20 mL/kg and <25 mL/kg, 1/3, 33%; ≥25 mL/kg and <30 mL/kg, 2/2, 100%; ≥30 mL/kg, 1/1, 100%).

**Table 5.** Incidence of AKI recovery in patients infused with the selected daily fluid volume.

	AKI recovery (%)		
Daily fluid infusion volume	Liberal fluid management^a^	Conservative fluid management^b^	*p*-Value
For 2 consecutive days from AKI onset (*N* = 90)			
≥5 mL/kg	23/56 (41%)	13/34 (38%)	0.828
≥10 mL/kg^c^	13/26 (50%)	23/64 (36%)	0.242
≥15 mL/kg	8/18 (44%)	28/72 (39%)	0.789
≥20 mL/kg	6/13 (46%)	30/77 (39%)	0.761
≥25 mL/kg	4/7 (57%)	32/83 (39%)	0.431
≥30 mL/kg	2/4 (50%)	34/86 (40%)	1.000
For 7 consecutive days from AKI onset (*N* = 90)			
≥5 mL/kg	19/41 (46%)	17/49 (35%)	0.287
≥10 mL/kg^c^	10/16 (63%)	26/74 (35%)	0.053
≥15 mL/kg	5/8 (63%)	31/82 (38%)	0.258
≥20 mL/kg	4/6 (67%)	32/84 (38%)	0.213
≥25 mL/kg	3/3 (100%)	33/87 (38%)	0.061
≥30 mL/kg	1/1 (100%)	35/89 (39%)	0.400

AKI: acute kidney injury.

Fisher’s exact test was performed to determine the *p*-values. The denominator denotes the number of participants.

^a^Daily fluid of a specified volume or more that is infused consecutively for a given period.

^b^Daily fluid of less than the specified volume infused for at least one day for a given period.

^c^Relisted from Table 2.

### Association between the duration of consecutive daily fluid infusion from AKI onset and AKI recovery in patients administered L-AMB

As shown in Table 6, we evaluated the incidence of AKI recovery for patients administered daily fluid volumes of ≥10 mL/kg or ≥25 mL/kg for the selected periods from the onset of AKI. Although the incidence of AKI recovery was found to be higher, though not significant, with the liberal fluid management group (≥10 mL/kg or ≥25 mL/kg) than with the respective conservative fluid management group for the evaluated periods, fluid infusions for 7 consecutive days yielded a relatively high incidence of AKI recovery (≥10 mL/kg, 63%; ≥25 mL/kg, 100%) than infusions for 2 consecutive days (≥10 mL/kg, 50%; ≥25 mL/kg, 57%) or 5 consecutive days (≥10 mL/kg, 50%; ≥25 mL/kg, 80%). Thus, despite the extremely limited number of subjects employed for these longer periods, extending the daily fluid infusion period over 7 days from AKI onset was not found to be associated with the incidence of AKI recovery in patients administered L-AMB.

**Table 6.** Incidence of AKI recovery in patients administered infused fluids for the selected periods.

	AKI recovery (%)		
Fluid infusion period from AKI onset	Liberal fluid management^a^	Conservative fluid management^b^	*p-*Value
Daily volum*e* ≥ 10 mL/kg			
For 2 consecutive days (*N* = 90)	13/26 (50%)	23/64 (36%)	0.242
For 5 consecutive days (*N* = 90)	12/24 (50%)	24/66 (36%)	0.331
For 7 consecutive days (*N* = 90)^c^	10/16 (63%)	26/74 (35%)	0.053
For 10 consecutive days (*N* = 90)	4/8 (50%)	32/82 (39%)	0.709
For 14 consecutive days (*N* = 80)	3/6 (50%)	30/74 (41%)	0.687
Daily volum*e* ≥ 25 mL/kg			
For 2 consecutive days (*N* = 90)	4/7 (57%)	32/83 (39%)	0.431
For 5 consecutive days (*N* = 90)	4/5 (80%)	32/85 (38%)	0.153
For 7 consecutive days (*N* = 90)	3/3 (100%)	33/87 (38%)	0.061
For 10 consecutive days (*N* = 90)	1/1 (100%)	35/89 (39%)	0.400
For 14 consecutive days (*N* = 80)	1/1 (100%)	32/79 (41%)	0.413

Fisher’s exact test was performed to determine the *p*-values. The denominator denotes the number of participants.

AKI: acute kidney injury.

^a^Daily fluid of a specified volume or more that is infused consecutively for a given period.

^b^Daily fluid less than the specified volume infused for at least one day for a given period.

^c^Relisted from Table 2.

### Changes in the level of serum creatinine in patients administered L-AMB who received daily consecutive fluid infusion

**Figure 2.**
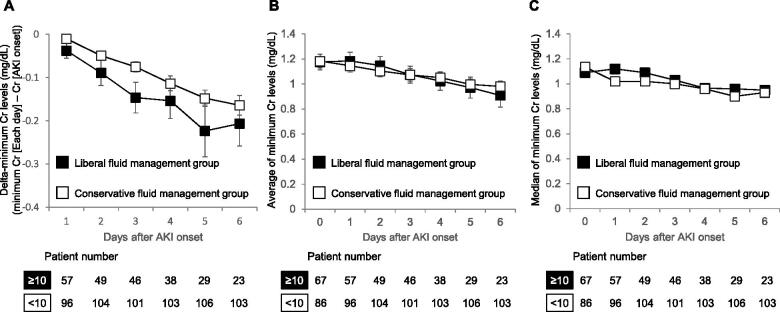
Change in minimum creatinine levels after the onset of AKI in the liberal and the conservative fluid management groups. (a) Delta-minimum Cr levels. On each day, the levels of minimum Cr were identified as the minimum value of Cr in the liberal fluid management group (≥10 mL/kg/day daily consecutive fluid infusion between AKI onset and each observation day) and the conservative fluid management group (<10 mL/kg/day fluid infusion for at least one day during the corresponding period). The levels of delta-minimum Cr were calculated as the difference between minimum Cr on each day and the Cr level on AKI onset. (b, c) Average (b) and median (c) of minimum Cr levels. As the unbiased levels of Cr on AKI onset were obtained until 6 days after AKI onset in the liberal fluid management group, we evaluated minimum Cr levels until 6 days after AKI onset. Error bars represent the standard error of the mean. Welch’s *t*-test was employed to compare the groups (a, b). AKI: acute kidney injury; Cr: creatinine; N: number.

Finally, changes in the minimum creatinine levels from AKI onset to each observation day were assessed for 153 patients; these patients had at least one creatinine record and no renal replacement therapy during the evaluation period. As described above, we compared the minimum creatinine levels from the day of AKI onset between the liberal and conservative fluid management groups to each of the first 6 days following AKI onset. As the unbiased levels of creatinine on AKI onset were obtained until 6 days after AKI onset in the liberal fluid management group, we evaluated minimum creatinine levels until 6 days after AKI onset. The liberal fluid management group have a decline, although not significantly, in delta-minimum creatinine (the difference between minimum creatinine on each day and the creatinine level on AKI onset) as well as the average and median of minimum creatinine for the first 6 days after the onset of AKI relative to corresponding conservative fluid management group (Figure 2(a–c)). On day 6 following AKI onset (i.e., after 7 days of consecutive fluid infusion, including the day of AKI onset), the minimum creatinine levels were reduced by 0.21 mg/dL for the liberal fluid management group and 0.16 mg/dL for the conservative fluid management group (Figure 2(a)).

## Discussion

In the present study, we retrospectively evaluated patients who developed AKI following treatment with L-AMB by using a dataset obtained from 345 medical facilities in Japan. In addition, we sought to determine the association between the interventions and AKI recovery. Previously, Yamazaki et al. suggested that intravenous fluid infusion one week before and after the initiation of L-AMB administration did not contribute to AKI recovery. Thus, we investigated the association between fluid infusion and AKI recovery when an infusion was administered before or from AKI onset (Table 2). Accordingly, we observed a higher, although not significant, incidence of AKI recovery for the liberal fluid management group (a 1.8-fold increase, 63 vs. 35%, *p* = 0.053). However, if limited to AKI stage 1 patients, the liberal fluid management group had a significantly higher recover rate (a 1.8-fold increase, 91 vs. 50%, *p* = 0.017) even after adjusting for confounding factors (OR: 10.135, 95% CI: 1.148–89.513, *p* = 0.037). On the other hand, though the number of subjects was limited, the liberal fluid management group did not have a higher incidence of AKI recovery for patients with AKI stage 2 or 3. This difference in prognosis may be due to the differences in the severity of underlying conditions including the use of catecholamine between AKI stage 1 patients (6/51, 12%) and AKI stage 2 or 3 patients (12/39, 31%, *p* = 0.034). The non-recovered patients in the liberal fluid management group may have partially recovered from AKI as they exhibited more reductions in minimum creatinine level from AKI onset though there were no significant differences (Figure 2). Furthermore, despite the limited number of subjects, a positive correlation was found between daily fluid volume for 7 consecutive days from AKI onset and incidence of AKI recovery (*p* = 0.043, Cochran–Armitage trend test), with an approximate 2.6-fold increase in the incidence of recovery from AKI of all stages for liberal fluid management group (≥25 mL/kg) relative to the conservative fluid management group. (Table 5). Extending the fluid infusion period beyond 7 days with a fixed volume did not result in a higher incidence of AKI recovery (Table 6), daily high-volume fluid infusion for 1 week from AKI onset may be associated with recovery from AKI.

To evaluate the association of consecutive fluid infusion over a period of time with AKI recovery, a liberal fluid management group was derived by selecting patients whose fluid infusion volumes were more than or equal to the threshold each day during a specified period and not patients whose mean fluid volume over the corresponding period was more than or equal to the threshold. In other words, the former definition specifically excludes patients that were intermittently infused with large volumes of fluid a few days out of the evaluation period and no fluid administration on the remaining days. We employed this definition of fluid management because the frequency of fluid infusion could be an important factor for AKI recovery. For instance, patients with a daily fluid infusion of ≥10 mL/kg/day for 7 consecutive days from AKI onset had an incidence of AKI recovery of 63% (10/16), whereas patients who received a mean fluid infusion volume ≥10 mL/kg/day for 7 days had an incidence of AKI recovery of 45% (14/31); the latter was however 27% (4/15) when patients administered daily fluid infusion were excluded. It should also be noted that fluids were confined to extracellular fluids, such as isotonic saline, which excluded parenteral nutrition with chloride as it was determined as a known risk factor for AKI [16].

The implication that daily high-volume fluid infusion from AKI onset may be associated with AKI recovery is consistent with standard interventions, such as salt loading against amphotericin B-associated nephrotoxicity [17]. However, such implications differ from the findings of Yamazaki et al., who reported that one week of fluid infusion before and after L-AMB administration initiation did not significantly improve the incidence of AKI recovery. This discrepancy between the two studies may be due to differences in the timing of fluid infusion, frequency of fluid infusion, and types of infused fluids.

Notably, if no interventions are found to be associated with AKI recovery after AKI onset, preventative measures will become more critical. Herein, liberal fluid management did not increase the incidence of recovery from stage 2 or 3 AKI; however, the risk factors associated with L-AMB-induced stage 2 or 3 AKI have been previously identified [12]. Special attention should be paid to patients with these risk factors: Hypokalemia before L-AMB therapy; prior treatment with angiotensin-converting enzyme inhibitors/angiotensin-receptor blockers or carbapenem; concomitant administration of catecholamines; and higher mean daily dose of L-AMB administration [12].

In patients with L-AMB-induced nephrotoxicity, adequate hydration and careful electrolyte supplementation could prevent or alleviate a reduction in eGFR or an increase in serum creatinine, without affecting on tubular toxicity [18,19]. The types of fluids may not have a remarkable effect on preventing or attenuating L-AMB-induced AKI. In this study, the incidence of AKI recovery was higher, although not significant, in patients receiving liberal fluid management with saline and/or balanced crystalloid solutions (8/11, 73%) than those receiving conservative fluid management with saline and/or balanced crystalloid solutions (7/17, 41%, *p* = 0.137), while the incidence of AKI recovery was similar between patients receiving liberal fluid management with saline alone (2/5, 40%) and those receiving conservative fluid management with saline alone (19/54, 35%, *p* = 1.000). Another group demonstrated that the combination of sodium bicarbonate and normal saline compared to normal saline alone appears to have no superiority in preventing or attenuating amphotericin B nephrotoxicity in patients with hematological malignancies [20].

In this study, the proportion of patients treated with vancomycin was higher in the group that did not recover from AKI (63%) than in the group that recovered from AKI (36%, *p* = 0.018). Furthermore, concomitant administration of vancomycin was negatively associated with recovery from AKI of all stages after adjusting for confounding factors (Supplementary Table 1) though it was not the case for stage 1 AKI. Vancomycin-induced renal toxicity may be due to oxidative effects on the proximal renal tubule, which results in renal tubular ischemia, or allergic interstitial nephritis [21], which is different from the mechanism underlying L-AMB-induced renal toxicity (i.e., tubular injury or renal vasoconstriction) [22,23]. Therefore, careful concomitant treatment with vancomycin may be needed to attenuate AKI in patients administered L-AMB.

Our study had several limitations. First, several data could not be obtained from the database, which limited the study design. Because the database did not include follow-ups after discharge or hospital transfer, the renal condition of patients was difficult to assess for a sufficiently long period. During the assessment of the incidence of AKI recovery, patients were excluded from the sample if they were discharged within 29 days or re-administered L-AMB within 30 days after AKI onset. Despite the inconsistent frequencies of serum creatinine measurements across patients, AKI recovery was only evaluated using the available records. Furthermore, we could not evaluate AKI by assessing reductions in urine volume, as this parameter could not be obtained from the database employed. Second, the generalizability of the findings presented herein requires further discussion. This is because the database did not contain patients admitted to university hospitals, where infectious disease experts were more likely to work or where the facilities had less than 200 beds. Additionally, in this study, patients who underwent renal replacement therapy (RRT), including dialysis and continuous renal replacement therapy, were excluded. Therefore, RRT was not considered an endpoint of AKI recovery. Accordingly, our study findings may not be fully representative of patients treated throughout Japan. Finally, further verification is required. Due to its retrospective design, this study could not prove a cause-effect relationship between fluid infusion and recovery from L-AMB-induced AKI. A clear dose-response effect regarding fluid volume or duration was not observed, which limits the enthusiasm regarding the findings. The introduction of liberal and conservative fluid management as dichotomous groups does not fully capture the complex and dynamic fluid infusion pattern over the evaluation period in real clinical settings. Confounders that increased fluid volumes may represent lower levels of serum creatinine, rather than a real representation of improved AKI recovery. The incidence of AKI following the L-AMB treatment initiation may not have been L-AMB-induced. Moreover, the sample size in several groups, especially patients administered with larger volumes of fluid infusion, is extremely small. Therefore, further studies with a large number of subjects from real-world databases and prospective studies are necessary to verify the results obtained herein.

In conclusion, 7 consecutive days of daily fluid infusion from the onset of AKI may be associated with recovery from stage 1 AKI in patients administered L-AMB. For those administered fluid infusions for 7 days from AKI onset, daily fluid volume was found to be positively correlated with the incidence of AKI recovery.

## Supplementary Material

Supplemental Material

## Data Availability

The data that support the findings of this study are available from Medical Data Vision Co., Ltd., but restrictions apply to the availability of these data, which were used under license for the current study, and so are not publicly available. The data, however, are available from the authors upon reasonable request and with permission from the Medical Data Vision Co., Ltd. (https://www.mdv.co.jp/).
