# Supplementary material for: Association between fluid infusions and the recovery from acute kidney injury in patients administered liposomal amphotericin B: a nationwide observational study
Source: Ren Fail. 2022 Feb 16;44(1):282–92. doi: 10.1080/0886022X.2022.2036618 (PMC8856109; doi:10.1080/0886022X.2022.2036618)
Supplement: Supplemental Material [file IRNF_A_2036618_SM3770.pdf]

Supplementary Table 1. Logistic regression analysis of the factors associated with AKI recovery from AKI of all stages in the liberal and the conservative fluid management groups

	Univariate regression		Multivariate regression		
Variables	OR (95% CI)	p-value	OR (95% CI)	p-value	VIF
Liberal fluid management[a], with (vs conservative fluid management[b])	3.077 (1.005-9.421)	0.049	2.381 (0.701-8.083)	0.164	1.037
Age, ≥65 years (vs <65 years)	1.724 (0.718-4.138)	0.223			
Sex, male (vs female)	1.126 (0.470-2.695)	0.790			
Comorbidities, with (vs without)					
Diabetes mellitus	0.961 (0.400-2.308)	0.929			
Congestive heart failure	1.099 (0.425-2.841)	0.846			
Catecholamine treatment, with (vs without)	0.509 (0.164-1.578)	0.242			
Hypokalemia (<3.5 mEq/L serum potassium, with [vs without])	0.338 (0.117-0.982)	0.046	0.489 (0.150-1.590)	0.234	1.062
Baseline eGFR, ≥60 mL/min (vs <60 mL/min)	0.331 (0.089-1.229)	0.099	0.530 (0.120-2.337)	0.402	1.091
L-AMB average daily dose (mg/kg/day, continuous value)	0.800 (0.484-1.322)	0.385			
Nephrotoxic drug treatment, with (vs without)					
Vancomycin	0.332 (0.138-0.798)	0.014	0.355 (0.143-0.882)	0.026	1.005
Aminoglycoside	0.438 (0.129-1.484)	0.185			
Carbapenem	0.769 (0.309-1.918)	0.574			
Immunosuppressants	0.625 (0.177-2.208)	0.465			
Diuretics	0.842 (0.340-2.084)	0.710			
ACE inhibitors/ARB	0.394 (0.117-1.325)	0.132			

ACE inhibitors/ARB, angiotensin-converting enzyme inhibitor/angiotensin receptor

blocker; AKI, acute kidney injury; CI, confidence interval; eGFR, estimated glomerular filtration rate; L-AMB, liposomal amphotericin B; OR, odds ratio; VIF, variance inflation factor.

Logistic regression analysis was conducted on patients with AKI of all stages using AKI recovery as the dependent variable. Fifteen independent variables associated with AKI or AKI recovery were subjected to univariate binomial logistic regression analysis. Variables with a p-value of <0.1 in univariate logistic regression analysis and liberal fluid management were subjected to multivariate logistic regression analysis. Comorbidities and fungal infections were identified using the corresponding ICD-10 codes that were registered in the month of L-AMB treatment initiation. Catecholamine and nephrotoxic drug treatment and hypokalemia were identified between 7 days before the day of L-AMB treatment initiation and the day before AKI recovery or the end day of the AKI recovery evaluation period (30 days after AKI, the day of discharge, death, or the day before L-AMB re-administration, whichever came first). The OR, 95% CI, and VIF were calculated.

[a]Daily fluid ≥ 10 mL/kg infused consecutively for 7 days from AKI onset

[b]Daily fluid < 10 mL/kg infused for at least one day for 7 days from AKI onset
